# Nearpod

**DOI:** 10.5195/jmla.2017.121

**Published:** 2017-01

**Authors:** Jorge E. Perez

Librarians who provide instruction, whether it be online or in-person, are always on the lookout for technology that enhances their presentations and makes their professional lives easier. More importantly, librarians seek technology to boost student engagement, promote collaborative experiences, assess student comprehension, and allow the freedom to create active classroom alternatives to the lecture-only format of yesteryear. Nearpod*,* a cloud-based application, allows instructors to create robust presentations on the fly in an easy-to-learn interface. The audience can view the presentation with any device, along with the presenter or self-paced, and interact during the lesson via activities.

Once you feel comfortable using the tool and shifting some of your personal instructional approaches, Nearpod can change the way you instruct. Nearpod provides lots of options to allow you to shift from lecture to individual or group activities and encourage students to create and co-teach. A presentation can be embedded in an online course for scaffolding lessons or flipping your classroom; in fact, Nearpod integrates with the Canvas learning management system (LMS). Even though the tool is marketed toward kindergarten through twelfth grade (K–12) teachers, educators at any level can take advantage of this tool’s features. According to the company website, higher education institutions are currently using this tool, including Stanford University, University of Maryland, University of Maine, University of South Carolina, Columbia University, and Boise State University [1].

Once you log in, you are presented with a dashboard that is easy to use and displays well on a variety of devices. The five main tabs are My Library, Explore, Join, Create, and Reports (Figure 1).

**Figure 1:**
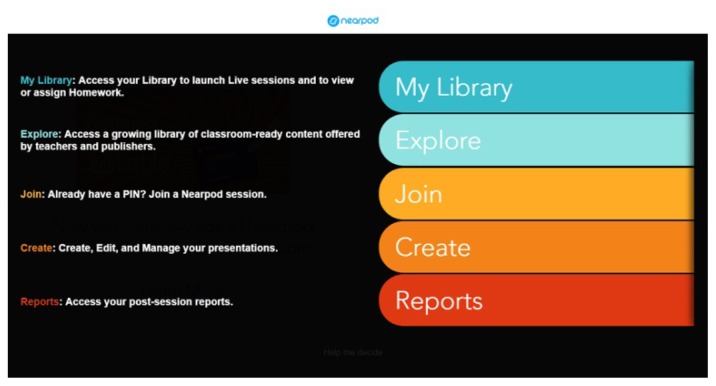
Nearpod dashboard view after clicking on Help Me Decide on the bottom of the screen

## MY LIBRARY

The My Library tab provides access to all of your lessons, whether created, downloaded for free, or purchased. To conduct a live lesson or share a self-paced learning object, you simply hover your cursor over the lesson and choose the desired option. In a live lesson, you invite participants to a particular presentation and, in real-time, lecture or provide an activity such as a poll question, quiz, or fill-in-the-blank question. You can share results with students, save the data for program assessment, or see if listeners are comprehending the lesson. This information can help you decide where to take the lecture, or students may ask pertinent lesson questions when viewing results data.

From the My Library tab, you can preview and edit your presentation; generate code to embed it in a learning management system, blog, or web page; export your presentation to portable document format (PDF); duplicate it; create folders in which to organize your presentations; and run reports. You can also generate an alphabetic code called a PIN that is unique to a specific lesson. You can then share this code via email or social media and pair with Google Classroom. PINs allow users to view instruction from any device, anywhere—a key feature that makes this tool worth the cost.

## EXPLORE

The Explore area provides access to Nearpod’s repository of premade presentations, some of which are free, which you can add to your library and customize for your needs. Unfortunately, the lessons currently available are relevant to K–12 students only.

## JOIN

Students use this tab to access a live or self-paced lesson, using the alphabetic code discussed previously. Students can complete self-paced lessons from anywhere and with any device—computer, phone, or tablet—or platform—iOS or Android—which permits a plethora of instructional approaches and classroom configurations.

## CREATE

Nearpod combines many features that have typically required multiple products. You can upload a slideshow, such as one created with Microsoft PowerPoint, or create a new slide show in Nearpod; embed a live Twitter feed to initiate comments and feedback during a live presentation; embed web links, audio files (.mp3, .wav, or .ogg), PDFs, and streaming video; and drag and drop files from a cloud storage system such as Google Drive or Dropbox. You can also embed a slideshow within a single slide. This feature is extremely helpful when you would like participants to review a lesson or follow a step-by-step process before moving on in the presentation. For example, a librarian can create a series of slides that emphasize each step to formulating a citation in American Medical Association style, or a math instructor can reiterate a lesson on the steps related to the order of operations rules.

Nearpod contains many tools to make lessons interactive, including open-ended questions, polls, quizzes, fill-in-the-blanks, memory test activities, and a Draw It tool that allows students to answer a question with words or by drawing an object. If students are using a tablet, they can draw with their fingers or a stylus. You can have students draw parts of the brain for a neurological or anatomy lesson or pose a question and have students write an answer freehand, “Final *Jeopardy*”–style. You can then show student responses—for example, poll answers or images created with Draw It—to the entire class. You can toggle back and forth by presenting slides, allowing students to complete activities on their own, and then assessing learning with activity results or feedback. As an instructor, you are able to see the results from all these interactive tools, tailor your presentation according to student needs, and track student comprehension in real time. In addition, Nearpod has a new tool called virtual fieldtrips. The instructor can embed famous areas in a “Google Street View”–like map display, where students can explore a place virtually with 360-degree views.

## REPORTS

Nearpod provides reports that can be shown to participants in real-time or provide data for the instructor to analyze or use for assessing students or course activities. Grades can be recorded in an electronic gradebook when students complete course activities. A report may assess student participation or show the ratio of correct answers. You can export the information as a PDF or in CSV format, compatible with Excel, for reviewing performance for a whole class or for a specific student. In addition, the system can automatically assign grades to an electronic gradebook. In today’s data-driven environment, these reports are vital for justifying a program, communicating to organization stakeholders, or improving a lesson. The appeal of Nearpod is that all these options are rolled into one in an easy-to-use format.

## PRICING

Nearpod offers 4 levels of pricing and features: Silver, Gold, School, and District. The Silver package is free and provides 50 megabytes (MB) of storage, access for a class of up to 30 participants, and basic features. The Gold package is $12 a month with 3 gigabytes (GB) of storage, access for up to 50 participants, and premium features. The School and District packages provide additional storage, accommodate larger class sizes, and include additional features as well as personalized training and support.

Librarians face many scenarios and obstacles when presenting in our subject area. We may be an invited or embedded instructor, and our students might have a dreadful stereotype of research and libraries. Also, librarians do not have the time to combine various free tools to create dynamic presentations. Nearpod can help librarians address these issues as well as avoid a plethora of technology issues associated with clickers and other tools used in interactive presentations. For a nominal fee, Nearpod provides a seamless experience for both instructors and students and allows a more active, collaborative learning environment.
